# Xiaozhang Tie Improves Intestinal Motility in Rats With Cirrhotic Ascites by Regulating the Stem Cell Factor/c-kit Pathway in Interstitial Cells of Cajal

**DOI:** 10.3389/fphar.2020.00001

**Published:** 2020-02-04

**Authors:** Qiang Zhao, Feng Xing, Yanyan Tao, Hongliang Liu, Kai Huang, Yuan Peng, Nianping Feng, Chenghai Liu

**Affiliations:** ^1^ Institute of Liver Diseases, Shuguang Hospital Affiliated to Shanghai University of Traditional Chinese Medicine, Shanghai, China; ^2^ Shanghai Key Laboratory of Traditional Chinese Clinical Medicine, Shanghai, China; ^3^ Department of Pharmaceutical Sciences, School of Pharmacy, Shanghai University of Traditional Chinese Medicine, Shanghai, China; ^4^ Key Laboratory of Liver and Kidney Diseases, Ministry of Education, Shanghai, China; ^5^ Shanghai Innovation Center of TCM Health Service, Shanghai, China

**Keywords:** Xiaozhang Tie (XZT), cirrhotic ascites, intestinal motility, interstitial cells of Cajal, stem cell factor (SCF)/c-kit.

## Abstract

We previously discovered that Xiaozhang Tie (XZT) was helpful for cirrhotic ascites, with obvious abdominal distention relief, suggesting that it may improve gastrointestinal (GI) motility. However, the underlying mechanisms of GI motility in cirrhotic ascites are unclear. Here, we aimed to discover explored the effect of XZT on GI motility in animal cirrhotic ascites and probed the action mechanism affecting GI motility by regulating the stem cell factor (SCF)/c-kit pathway in interstitial cells of Cajal (ICCs) and GI hormones. First, rat models of cirrhotic ascites were developed and then divided randomly into the following three subgroups: model control, XZT group, and mosapride group. The efficacy of XZT on treating cirrhotic ascites was evaluated on the basis of ascites weight and volume, 24 h urine volume, and feces water content. GI motility of the cirrhotic model, intestine propulsion, and gastric residue were detected using the migration distance of ink *in vivo*, and the frequency of contraction and tension of isolated gastric and jejunal muscle strips were measured after incubation with XZT extracts. Serum GI hormone content, including motilin (MTL), substance P (SP), somatostatin (SS), and vasoactive intestinal polypeptide were assayed. Subsequently, ICCs were isolated from jejunum, and primarily cultured ICCs were incubated with and without XZT and SCF. The cell vitality of the ICCs was measured. A whole-cell patch recording technique was used to record the current of K^+^ and Na^+^ channels in the ICC membrane. Expressions of c-kit/p-c-kit, p-Akt, p-STAT3, and p-Erk1/2 were detected *in vivo* and *in vitro*. The results revealed that XZT significantly reduced ascites weight and increased urine volume and fecal water content in model rats. XZT promoted intestinal motility and increased MTL level but reduced SP and SS levels. It enhanced the current of Na^+^ and K^+^ in ICCs and improved c-kit expression and signaling mediator phosphorylation in SCF/c-kit, which was inhibited by imatinib *in vitro* and downregulated in model rats *in vivo*. Our study concluded that XZT reduced the amount of ascites and improved intestinal motility in cirrhotic rats, which may be associated with its effect on ascites and was involved in the mechanisms regulating the SCF/c-kit signaling pathway in ICCs and improving gastrointestinal hormone secretion.

## Introduction

Ascites is the most common clinical manifestation of cirrhosis (Runyon and Committee, 2009) and is an indicator that cirrhosis has developed from the compensation stage to the decompensation stage. It not only affects the quality of life of patients but is also closely related to other complications of cirrhosis, such as endotoxemia, spontaneous bacterial peritonitis, hepatorenal syndrome, and gastrointestinal dysfunction. Over half of compensated cirrhosis cases develop ascites within 10 years, with 1- and 5-year mortality rates of approximately 15% and 50%, respectively, for patients with cirrhotic ascites (Planas et al., 2006; Hsu and Huang, 2013). Thus, methods of managing cirrhotic ascites have great clinical significance.

Two therapeutic strategies for cirrhotic ascites are currently used. One is drug therapy, including diuretics and human serum albumin. Another is invasive therapy, such as peritoneal paracentesis and autoinfusion of concentrated ascites. However, the use of diuretics and large-volume paracentesis are likely to cause electrolyte disturbances and circulatory dysfunction, and are less effective for treating refractory ascites. Thus, a suitable method for the management of cirrhotic ascites, refractory ascites in particular, remains to be identified, and new treatments are required.

In Chinese medicine, external patching therapy for cirrhosis and ascites through application of a patch with drugs such as euphorbia gansui, mirabilite, and euphorbia on the body has a history extending thousands of years. This external patching therapy usually involved mixing drug powder with vinegar and pasting the mixture onto the patient’s navel. In our previous work, we designed a new formula named Xiaozhang Tie (XZT), composed of dahuang (*Rheum palmatum L.*), laifuzi (*Raphanus sativus L.*), gansui (*Euphorbia kansui T.N. Liou ex T.P. Wang*), chenxiang [*Aquilaria sinensis (Lour.) Gilg*], dingxiang (*Eugenia caryophyllata Thunb.*), bingpian (*borneolum syntheticum*), and shexiang (*artificial Moschus*), and developed a modern pharmaceutical dose. Our clinical trials indicated that XZT increased urine output and reduced the amount of ascites in patients with cirrhotic ascites without obvious side effects. In particular, after the initial application, it increased exhaust and defecation, improved appetite, and alleviated abdominal distention (Xing et al., 2012), suggesting that it could improve patients’ gastrointestinal motility by acting on ascites.

Studies have demonstrated that patients with cirrhotic ascites often have prolonged gastrointestinal transit time and gastrointestinal motility disorders, such as indigestion and flatulence. In addition, bacterial proliferation and translocation may occur in the intestinal tract of patients with cirrhotic ascites as a result of gastrointestinal motility dysfunction and portal hypertension, thereby increasing the amount of ascites and leading to higher susceptibility to other complications such as endotoxemia, spontaneous bacterial peritonitis, and hepatorenal syndrome (Chander Roland et al., 2013). Therefore, the discovery of gastrointestinal motility disorder has key clinical implications for patients with cirrhotic ascites; it can not only improve quality of life by alleviating abdominal distention, constipation, and other symptoms but also reduce the amount of ascites by promoting urinary and fecal excretion (Zhang et al., 2017). The relationship between gastrointestinal motility and cirrhotic ascites is not fully understood, however, and the action mechanism of effective agents on ascites with respect to gastrointestinal motility remains unclear.

In the current study, we attempted to answer the following two questions through *in vivo* and *in vitro* experiments: (1) whether the XZT effect on reducing the amount of cirrhotic ascites is associated with improved gastrointestinal motility and (2) if so, what the action mechanism of XZT is in regulating gastrointestinal motility in cirrhotic ascites.

## Materials and Methods

### Drugs

Mosapride citrate (License No. H19990317) was provided by Lunan-beite Pharmaceutical Co., Ltd. (Shandong, China). Imatinib Mesylate (Art. No. T1621) was purchased from TargetMol (Boston, MA, USA). XZT, and blank poultices were provided by Changshu Leiyunshang Pharmaceutical Co., Ltd. (Jiangsu, China).

### Compositional Analysis of XZT

The formula for XZT (one dose): 1.0 g of dahuang, 1.0 g of laifuzi, 1.0 g of gansui, 0.2 g of chenxiang, 1.0 g of dingxiang, 0.04 g of borneolum syntheticum, and 0.004 g of artificial Moschus.

The manufacturing procedures for the XZT and blank poultices were detailed by Xing et al. (2012). XZT was extracted through ultrasonication in an aqueous solution of methanol, and essential oils were obtained using a hydrodistillation method. Subsequently, the XZT extract was characterized using a Waters Acquity Ultra-Performance LC-Synapt G2 Q/TOF system (Waters Corporation, Milford, MA, USA). The composition of XZT extract includes more than 50 ingredients, such as gallic acid, desulfo-glucoraphanin, and glucoraphenin. Additional details regarding the extraction and **Supplementary Methods** were provided by Zhang et al. (2019).

### Reagents

*In vivo*, a cirrhotic rat model complicated with ascites was established with CCl_4_ (Cat. No. 10006428) and olive oil (Cat. No. 69018028) obtained from Sinopharm Group Co., Ltd. (Shanghai, China). Enzyme-Linked Immunosorbent Assay (ELISA) kits of stem cell factor (SCF) (Art. No. YX-190306R), p-Akt (Art. No. 011120R), p-c-kit (Art. No. 110920R), p-STAT3 (Art. No. YX-012003R), and p-ERK1/2 (Art. No. 181102R) were purchased from Pepro Tech Inc. (Su Zhou, China), and the kits of substance P (SP) (Art. No. 10171), somatostatin (SS) (Art. No. XF-100), motilin (MTL) (Art. No. XF-094), and vasoactive intestinal polypeptide (VIP) (Art. No. XF-10162) were offered and their indexes tested by Shanghai Xinfan Biotechnology Co., Ltd. (Shanghai, China). The rabbit antihuman polyclonal c-kit primary antibody (Art. No. SC-365504) was purchased from CST Inc. (Shanghai, China). The rabbit SABC immune-histochemical kit (Art. No. SA1022) and DAB color development kit (Art. No. AR1022) were purchased from Boster Bio-Engineering Limited Company (Wuhan, China).

*In vitro*, polyclonal primary antibodies of akt (Art. No. 4691), stat3 (Art. No. 12640), erk1/2 (Art. No. 4695), p-Akt (Art. No. 4060), p-STAT3 (Art. No. 98543), and p-ERK1/2 (Art. No. 9101) were purchased from Abcam (Cambridge, UK) to determine the expression levels of the related proteins in interstitial cells of Cajal (ICCs) through western blotting.

### Animals and Experimental Design

In this experiment, 80 male Sprague-Dawley (SD) rats weighing 170±10 g were purchased from Shanghai SLAC Laboratory Co. Ltd. (Shanghai, China). All rats were housed in an animal room under standard conditions, with a temperature of 22°C, humidity of 55%, 12 h light dark cycle, and free access to food and water. The study was carried out in accordance with the Committee on the Care and Use of Live Animals for Teaching and Research of the Shanghai University of Traditional Chinese Medicine, and the procedures were performed according to the guidelines of the committee.

First, 80 male SD rats were randomly divided into two groups, a normal control group (n  =  10) and a model group (n  =  70). To make rats cirrhotic with ascites, rats were injected intraperitoneally with 20% CCl_4_ for the first week, 30% CCl_4_ for the second week, and then 40% CCl_4_ at a dose of 2 ml·kg^−1^ twice a week until the cirrhotic model was successfully established. The rats in the control group were injected with the same volume of pure olive oil. Eventually, the ascites model of cirrhosis was established successfully in 29 rats, and these rats were randomly divided into three groups as follows: a model group (*n*  =  9), an XZT group (*n*  =  8), and a mosapride group (*n*  =  8). The remaining four mice were used for *in vitro* gastrointestinal electrophysiological testing. The body weight and urine output volume in each group were measured and recorded on a daily basis for treatment assessment. Subsequently, rats in the test group were administered an umbilical compress with XZT at a daily dose of 2.25 cm^2^ for 1 week, while those in positive control group were treated with mosapride citrate orally at dose of 2 mg·kg^−1^ for 1 week. On their last day in metabolic cages, all rats were deprived of food for 12 h, but water was allowed. The feces were collected and measured. The wet feces were dried in an oven at 60°C for 24 h. The fecal water content was calculated using the following calculation formula: [wet weight (g) − dry weight (g)]/wet weight (g) × 100%. After a 7-day intervention and observation period in metabolic cages, all rats were intragastrically administered nutritious semisolid paste containing ink to determine the propulsive rate of the small intestine. After 30 min, the rats were subjected to anesthesia and laparotomy, and serum and liver samples were harvested. The small intestinal tract from the pylorus to the ileocecal valve was removed, and the distance from the pylorus to the front of the ink was measured as the migration distance of the ink. The following formula was used to calculate the ink propulsion rate: ink propulsion rate (%) = migration distance of ink/whole length of the small intestine × 100%.

### Immunohistological Analysis of c-kit in Jejunum Sections

A 1 cm segment of jejunum at a distance of 1 cm from the duodenum was taken for immunohistochemical analysis. Jejunum tissues were fixed with 10% formalin, embedded in paraffin, cut into 4 µm sections for staining with rabbit antihuman polyclonal c-kit primary antibody (Art. No. SC-365504), and visualized with the rabbit SABC immunohistochemical kit (Art. No. SA1022) and DAB color development kit (Art. No. AR1022). An Olympus DP71 digital charge-coupled microscope device was used to collect positive images, and Image-Pro Plus 6.0 software was used for semiquantitative analysis of the c-kit positive expression area of jejunum tissue.

### Measurements of Gut Hormones in Serum

Serum levels of gut hormones such as MTL, SP, SS, and VIP were detected through radioimmunoassay with commercial kits purchased from Shanghai Xin Fan Biotechnology Co., Ltd. (Shanghai, China).

### ELISA

Levels of SCF (Art. No. YX-190306R), p-c-kit (Art. No. 110920R), p-STAT3 (Art. No. YX-012003R), p-Akt (Art. No. 011120R), and p-ERK1/2 (Art. No. 181102R) in serum were detected according to instructions provided by SCIGE Biotechnology Co., Ltd. (Shanghai, China) for using commercial ELISA kits.

### Isolation and Identification of ICCs

In this experiment, 10 g of germ-free 3-day-old C57/BL6 neonatal mice were decapitated, and their jejunum was separated and cut into 1 mm^3^ sections. The tissue fragments of intestine were washed with phosphate-buffered saline (PBS) and digested with a trypsin–EDTA solution (sterile PBS containing 0.25% trypsin and 0.02% EDTA under pH 8.0) at 37 °C for 1 h. After a series of filtration, centrifugation, and precipitation, the cells were seeded in culture flasks and cultured in an incubator at 37 °C in a 5% CO_2_ atmosphere. After 24 h, the medium was carefully changed. The cells were incubated with c-kit antibody (1 μg/1 × 10^6^ cells) for 30 min. After PBS resuspension and centrifugation for 5 min, the cells were incubated with goat antirabbit IgG H & L for 30 min at 22°C. Cell purity was detected using a flow cytometer.

### Cell Culture

The identified primary ICCs were cultured in a 1640 medium supplemented with 10% fetal bovine serum, 100 units/ml of penicillin, and 100 units/ml of streptomycin. All the cells were cultured in a humidified incubator at 37°C in a 5% CO_2_ atmosphere.

### Cell Viability Assay

The extracts of XZT, SCF, and imatinib were initially dissolved in sterile PBS as a stock solution. To confirm a suitable concentration for the drugs on ICCs *in vitro*, ICCs were cultured in 96-well plates with the three drugs at the concentrations of 0, 0.1, 1, 10, 50, and 100 ng/ml. After a 48 h treatment, cell viability assays were performed using the Cell Counting Kit 8 (CCK8) (Dojindo Laboratories, Kumamoto, Japan), which identified the most suitable concentrations of the drugs on ICCs to be 100 ng/ml for SCF, 100 ng/ml for imatinib, and 50 ng/ml for XZT.

To evaluate the effects of different drugs and drug combinations on the cell viability of primary ICCs, the cells were divided into six groups and incubated with the most suitable concentrations of the drugs and with drug combinations (**Table 1**) for 48 h, and CCK8 was employed to evaluate the viability of ICCs.

**Table 1:** Grouping and treatment of ICCs.

	Blank	Imatinib	Imatinib and XZT	Imatinib and SCF	XZT	SCF
SCF	−	−	−	+	−	+
Imatinib	−	+	+	+	−	−
XZT	−	−	+	−	+	−

ICCs, interstitial cells of Cajal; XZT, Xiaozhang Tie; SCF, stem cell factor.

### Electrophysiology of ICCs

To study the effects of XZT on the electrophysiology of ICCs, a whole-cell patch clamp was used to detect K^+^ and Na^+^ channel currents in the ICC membrane. The resistance of the glass pipette was 2–4 MΩ, and whole-cell recording mode was established using a suction after a gigaohm (KMΩ) seal was obtained. For the detection of Na^+^ channel currents, the following reagents were used: 140 mM NaCl, 0.1 Mm CdCl_2_, 20 mM TEA-Cl, 5 mM CsCl, 2 mM CaCl_2_, 1 mM MgCl_2_.6H_2_O, 10 mM HEPES, and 10 mM glucose in the extracellular solution and 10 mM NaCl, 10 mM HEPES, 130 mM CsF, and 10 mM EGTA in the internal solution. In the extracellular solution, a sodium hydroxide solution was used to adjust the pH to 7.4 and the osmolality to 300–310 mOsm, and a cesium hydroxide solution was used in the pipette solution to adjust the pH to 7.2 and osmolality to 295–300 mOsm. The holding potential was set at −60 mV, and the current of Na^+^ channel was brought out by a 0 mV voltage for 20 ms every 2 s. To avoid influencing the detection of Na^+^ channel currents, the components TEA, Cs^+^, and Cd^2+^ in the extracellular solution were applied to block other ion channels. For the detection of the K^+^ channel current, the extracellular solution contained 135 mM NaCl, 5.4 mM KCl, 0.2 mM CdCl_2_, 1 mM CaCl_2_, 1 mM MgCl_2_, 0.33 mM NaH_2_PO_4_, 5 mM HEPES, and 5 mM glucose, whereas the internal solution consisted of 140 mM KCl, 1 mM MgCl_2_, 5 mM HEPES, 10 mM EGTA, and 2 mM Na_2_ATP. Similarly, a sodium hydroxide solution was added to the extracellular solution to adjust the pH to 7.4 and osmolality to 300–310 mOsm, and potassium hydroxide was used in a pipette solution to adjust the pH to 7.2 and osmolality to 295–300 mOsm. ICCs were claimed at the holding potential of −40 mV, and the experimental potential stepped to +50 mV from −120 mV by + 20 mV increments for 300 ms at a frequency of 0.2 kHz.

### Western Blotting

ICCs were lysed in RIPA lysis buffer containing 150 mmol/L NaCl, 1% NP-40, 0.1% SDS, 50 mmol/L Tris–HCl pH 7.4, 1 mmol/L EDTA, 1 mmol/L PMSF, and 1 × complete mini. Lysates were centrifuged at 10,000 × g at 4 °C for 15 min to collect lysate. The protein concentration was quantified using a bicinchoninic acid protein assay. Under denaturing and nonreducing conditions, 50 µg of total protein was separated by 10% SDS gel electrophoresis and then transferred to immunobilon-p transfer membranes. Membranes were blocked with 5% nonfat milk in TBST (20 mmol/L Tris–HCl, pH 7.5, 150 mmol/L NaCl, 0.1% Tween 20) at room temperature for 1 h and incubated with c-kit (Art. No. SC-365504), Akt (Art. No. ab4691), STAT3 (Art. No. ab12640), ERK1/2 (Art. No. ab4695), p-Akt (Art. No. ab4060), p-STAT3 (Art. No. ab98543), and p-ERK1/2 (Art. No. ab9101) primary antibodies overnight at a temperature of 4°C. After washing with TBST, blots were incubated with a horseradish-coupled secondary antibody in wash buffer. Signals were developed and immunoreactive bands were visualized using an ECL kit (Upstate Biotechnology, Lake Placid, NY, USA) according to the manufacturer’s instructions and were quantified using a Chemi-Doc image analyzer (Bio-Rad, Hercules, CA, USA).

### Function of Isolated Gastrointestinal Muscle

The present study involved pre-experiments and formal experiments. A cirrhotic ascites model was established in rats as described. Laparotomy was carried out as soon as the four model rats and two normal rats were decapitated. Their stomachs and jejunums were separated and placed in a Tyrode’s solution bath. The solution was set at a temperature of 37°C, and a mixed gas of 95% O_2_ + 5% CO_2_ was passed into the bath through an L-shaped hook. Contents in the gastrointestinal tract were cleared and the intestinal villus, mesentery, blood vessels, and adipose tissues affiliated with gastrointestinal tissues were carefully removed. The gastrointestinal segments were cut into 2 × 5 mm^2^ muscle strips and separately placed in Tyrode’s solution baths. The upper ends of the muscle strips were connected to a tension transducer, and the lower ends were fixed to the L-shaped hook. The tension transducer was connected to a BL-420 biology function laboratory system (Chengdu Instruments Factory Co., Ltd., Sichuan, China), and the contractile activities of the muscle strips were recorded.

In the pre-experiment, an XZT extract solution was added to the bath separately at different concentrations (0.001, 0.1, 1, 10 , 50 , and 100 µg/ml), and the muscle-contraction curves were recorded. Because the XZT extract solution at the concentrations of 0.001, 0.1, and 1 µg/ml showed no obvious effect on tension or contractile frequency of the gastrointestinal muscle *in vitro*, solution concentrations of 10, 50, and 100 µg/ml were selected for the formal experiment, in which the steps were repeated with escalating concentrations of 10, 50, and 100 µg/ml of the XZT extract solution. The tension and contraction frequency of gastrointestinal muscle obtained from healthy rats and those with cirrhotic ascites were both measured and recorded before and after XZT treatment.

### Statistical Analysis

All data were analyzed with PASW Statistics 18 software. Differences between groups were assessed using nonparametric one-way analysis of variance, and *p* < 0.05 was considered statistically significant. Values in the text are presented as the mean ± SD.

## Results

### XZT Improved Urine Volume and Fecal Water Content in Cirrhotic Ascites in Rats

**Figure 1:**
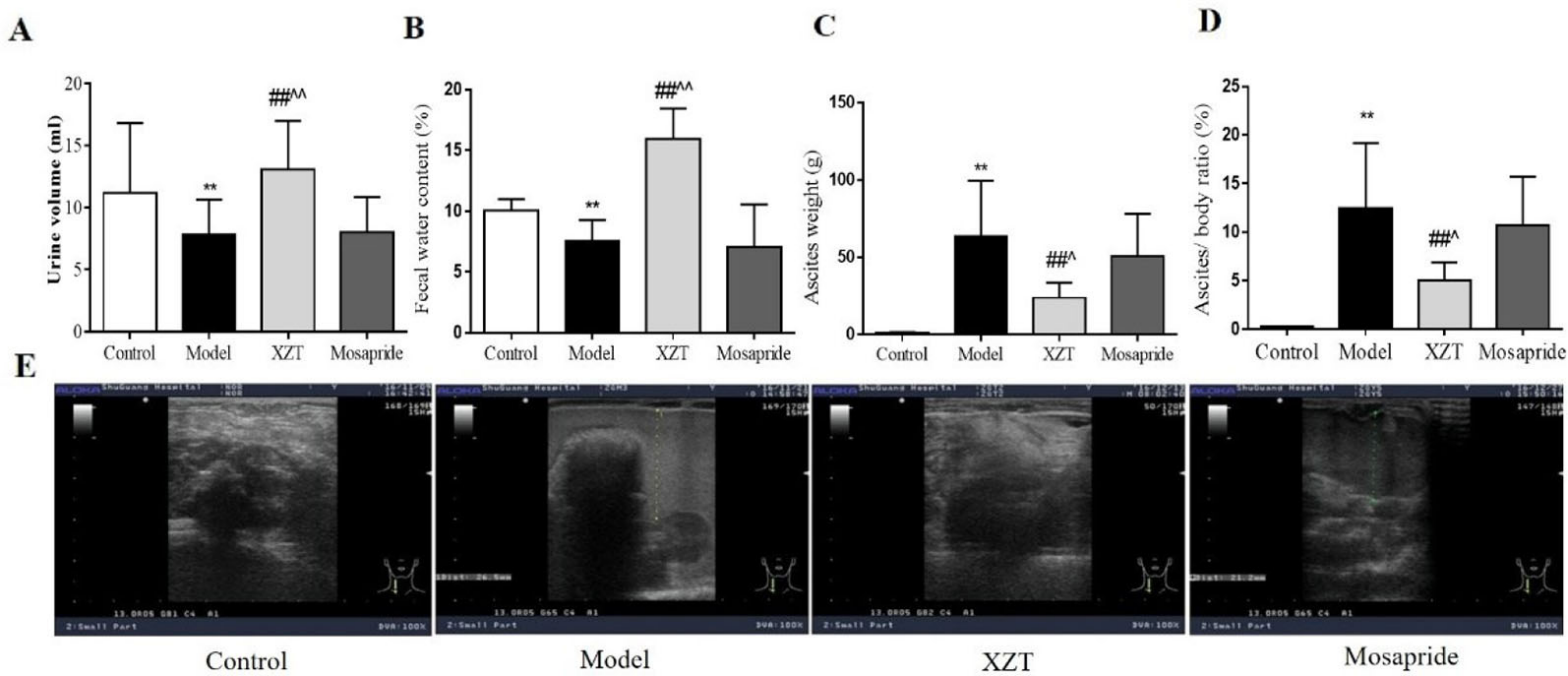
Xiaozhang Tie (XZT) increased the urine volume and fecal water content in rats with cirrhotic ascites. Cirrhosis with ascites was established as follows. A total of 70 rats were injected intraperitoneally with a 20% CCl_4_–olive oil solution for the first week, 30% CCl_4_ for the second week, then 40% CCl_4_ at a dose of 2 ml·kg^−1^ twice a week until B ultrasound confirmed the ascites model of cirrhosis. Eventually, cirrhotic ascites was successfully induced in 29 rats, and the model rats were randomly divided into the following three groups: model group (n = 9), XZT group (n = 8), and mosapride group (n = 8). The remaining model rats were subjected to further electrophysiological testing of the gastrointestinal tract (n = 4). In the normal control group, 10 rats were injected with the same volume of pure olive oil. All rats were transferred to metabolic cages. Rats in the XZT group were administered XZT in the umbilical region at a daily dose of 2.25 cm^2^ once a day for 7 days, and those in the positive control group were orally administered mosapride citrate tablets at a dose of 2 mg·kg^−1^ once a day for 7 days. After a 7-day intervention and observation period in metabolic cages, urine and feces were collected over a period of 24 h before the experiment concluded. Body weight and ascites weight were recorded during the execution of the rats. **(A)** 24 h urine volume. **(B)** Wet feces were dried in an oven at 60°C for 24 h. Fecal water content was calculated using the following calculation formula: [wet weight (g) − dry weight (g)]/wet weight (g) × 100%. **(C)** The weight of ascites and **(D)** ratio of ascites weight to body weight was calculated. **(E)** The amount of ascites was roughly assessed through B ultrasound. Each bar represents the mean ± SD. **p < 0.01 compared with the normal group; ^##^ *p *<0.01 compared with the model group; ^^^ *p *< 0.05 and ^^^^ *p *< 0.01 compared with the mosapride group.

Fluid dark areas in the abdomen were detected through B ultrasound in the 29 model rats. High ascites/weight ratio accompanied by reduced 24 h urine volume and fecal water content were observed in the cirrhotic rats, and all pathological phenomena were effectively reversed by XZT but not by mosapride (**Figures 1A–E**). In the model rats, serum transaminases levels increased markedly significantly, and a large amount of collagen was deposited as a result of the formation of pseudo lobules in the interstitial space of liver tissue sections. Neither XZT nor mosapride had an effect on serum liver function parameters or histological changes in the cirrhotic model rats (data not shown).

### XZT Improved Intestinal Motility of Rats With Cirrhosis And Ascites *In Vivo* and *In Vitro*

**Figure 2:**
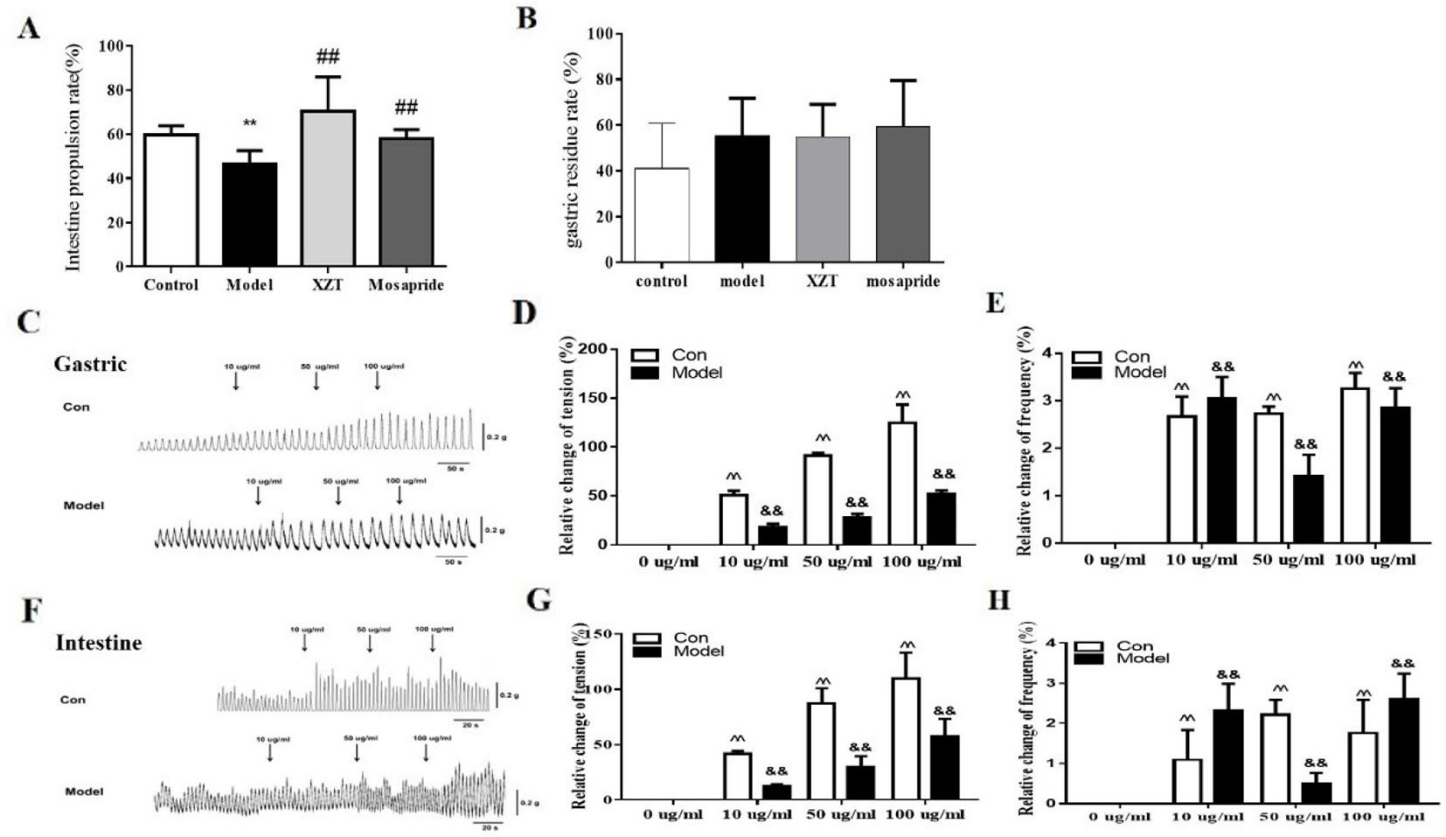
XZT improved intestinal motility of rats with cirrhosis and ascites *in vivo* and *in vitro*. The small intestine propulsion ratio **(A)** and gastric residual ratio **(B)** were determined with ink *in vivo*. After a 7-day intervention, all rats were intragastrically administrated nutritious semisolid paste (5.0 g of sodium carboxymethyl cellulose, 8.0 g of milk powder, 4.0 g of sucrose, 4 g of starch, 2.0 ml of carbon ink, and 125 ml of distilled water) containing ink to determine the gastric residual ratio and intestinal propulsion ratio. The rats were given 1 ml/kg semisolid nutritional paste and then deprived of food for 12 h before sacrifice; 30 min later, the rats were anesthetized with 3% pentobarbital sodium. The stomachs of the rats were cut off at the distant part of the cardia and duodenum at the lower end of the esophagus, and the small part above the intestine was removed and straightened. The formula for calculating gastric residual ratio was as follows: (total weight of stomach − net weight of stomach)/semisolid nutrition paste weight × 100%. The movement distance in the small intestine of the paste was measured using a soft ruler. The formula used for calculation the small intestine propulsion ratio is as follows: migration distance of ink/whole length of small intestine × 100%. **(C**–**H)** *In vivo*, two normal control rats and four rats with cirrhotic ascites were sacrificed, and the jejunums and stomachs with appropriate sizes were used to measure gastrointestinal muscle strip tension and contraction frequency. First, XZT extract was screened and its optimal concentrations were confirmed to be from 10 to 100 µg/ml (data not shown). The frequency and tension of the gastric and jejunal muscle strips were recorded using a biosignal collection system **(C**, **G)**. **p < 0.01 compared with the control (normal) group; ^##^ *p *< 0.01 compared with the model group; ^^^^ *p *< 0.01 compared with the normal group muscle strips; ^&&^ *p *< 0.01 compared with the model control group muscle strips.

In vivo, both XZT and mosapride considerably increased the small intestinal propulsion rate, which was restricted by model establishment (**Figure 2A**), and neither affected the gastric residue ratio (**Figure 2B**). *In vitro*, gastric and jejunal muscle strips isolated from two normal and four cirrhotic ascites rats were incubated with XZT extract at different concentrations to screen for the optimal concentration. It was found that 10–100 µg/ml of XZT obviously increased the frequency and tension of isolated gastric and jejunal muscle contraction (data not shown). Thus, muscle strips were treated with XZT extract at concentrations of 10, 50, and 100 µg/ml (**Figures 2C–H**). The results suggested that XZT extract at these three concentrations increased the frequency and tension of isolated gastric and jejunal muscle contraction in both the control and model groups. A biosignal collection system was used to record the experiment and process the results (**Figures 2C, F**), which underwent semiquantitative analysis (**Figures 2D**, **E**, **G**, and **H**).

### XZT Regulated the Dysfunction of Gastrointestinal Hormones in Cirrhotic Rats

**Figure 3:**
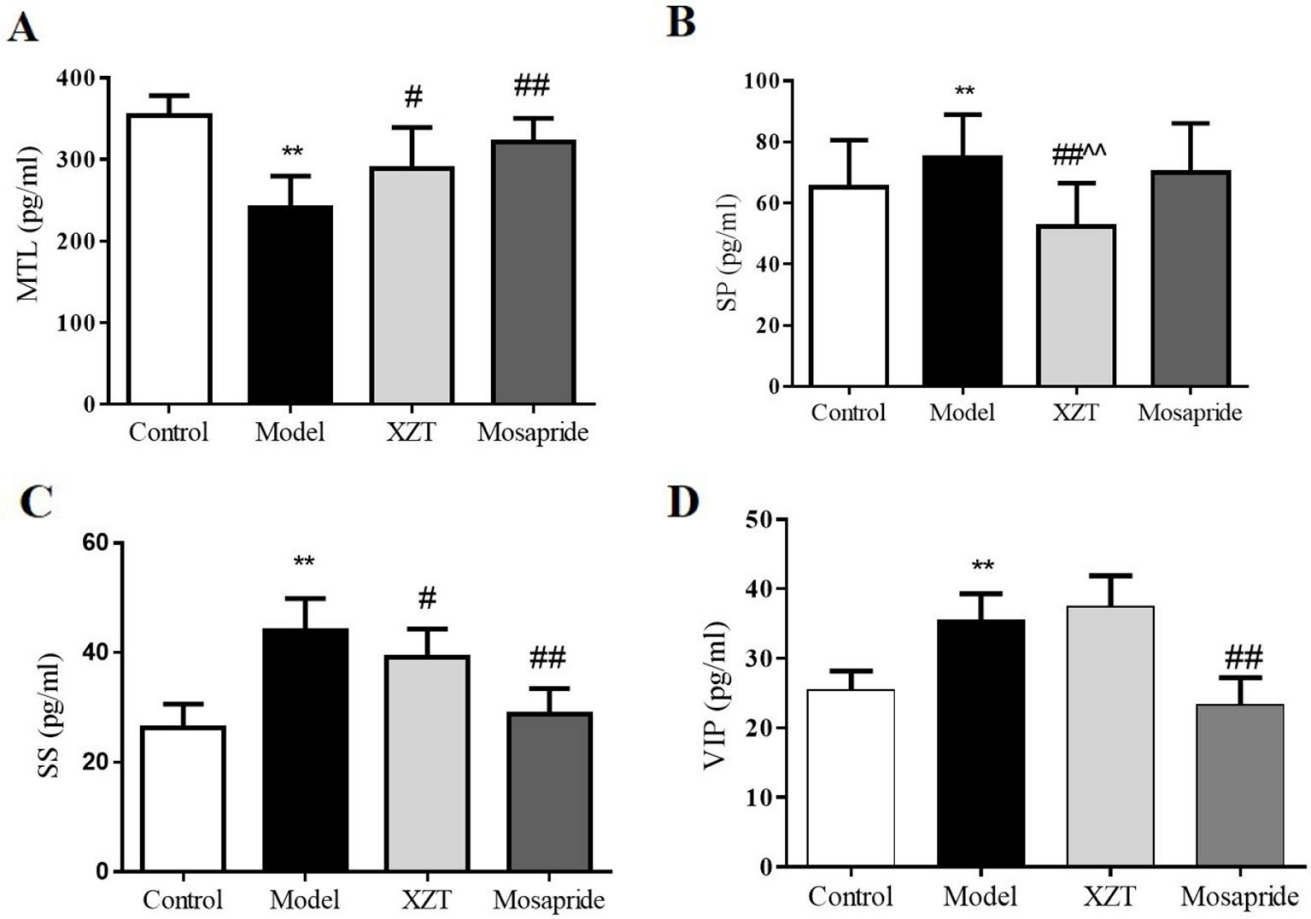
XZT regulated the dysfunction of gastrointestinal hormones of rats with cirrhosis and ascites. *In vivo*, gastrointestinal hormones of **(A)** MTL, **(B)** SP, **(C)** SS, and **(D)** VIP in serum were measured using a radioimmunoassay technique. **p < 0.01 compared with the control (normal) group; ^#^ *p *< 0.05 and ^##^ *p *< 0.01 compared with the model group; ^^ p < 0.01 compared with the Mosapride group.

Gastrointestinal hormone levels in rat serum were tested through radioimmunoassay. As can be seen in **Figure 3**, model establishment significantly reduced MTL level (**Figure 3A**) but notably elevated SP (**Figure 3B**), SS (**Figure 3C**), and VIP (**Figure 3D**) levels. The gastrointestinal hormone levels of MTL, SP, and SS were considerably reversed after the administration of XZT; mosapride upregulated the level of MTL and downregulated SS and VIP levels.

### XZT Regulated SCF/c-kit Pathway in ICCs

**Figure 4:**
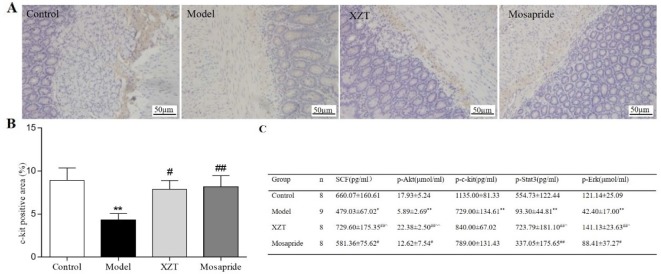
XZT regulated the stem cell factor (SCF)/c-kit pathway in interstitial cells of Cajal (ICCs) *in vivo*. **(A)** A 1 cm segment of jejunum at a distance of 1 cm from the duodenum was taken for kit immunostaining, then a full slide of immunostained tissue was digitally scanned. Semiquantitative analysis was conducted with Image-Pro Plus 6.0 software. **(A)** Expression of ICCs in jejunal tissues according to immunohistochemical staining (×200). **(B)** Semiquantitative analysis of the positive staining rate (%) of c-kit in jejunal tissues. **(C)** Serum SCF level and phosphorylation of the downstream factors in the SCF/c-kit signaling pathway of small intestine tissues in rats were calculated using Enzyme-Linked Immunosorbent Assay (ELISA). ^*^ *p *< 0.05, **p < 0.01 compared with the blank group; ^#^ *p *< 0.05, ^##^ *p *< 0.01 compared with the mosapride group.

Immunohistochemical staining revealed that XZT upregulated the expression of c-kit in jejunal tissues in cirrhotic ascites rats (**Figures 4A, B**). The SCF level and phosphorylation of c-kit, Akt, Stat3, and Erk1/2 in rat serum were measured using ELISA *in vivo* (**Figure 4C**), and XZT upregulated the expressions of p-c-kit, p-Akt, p-STAT3, and p-ERK1/2. The effect of XZT was similar to that of mosapride.

**Figure 5:**
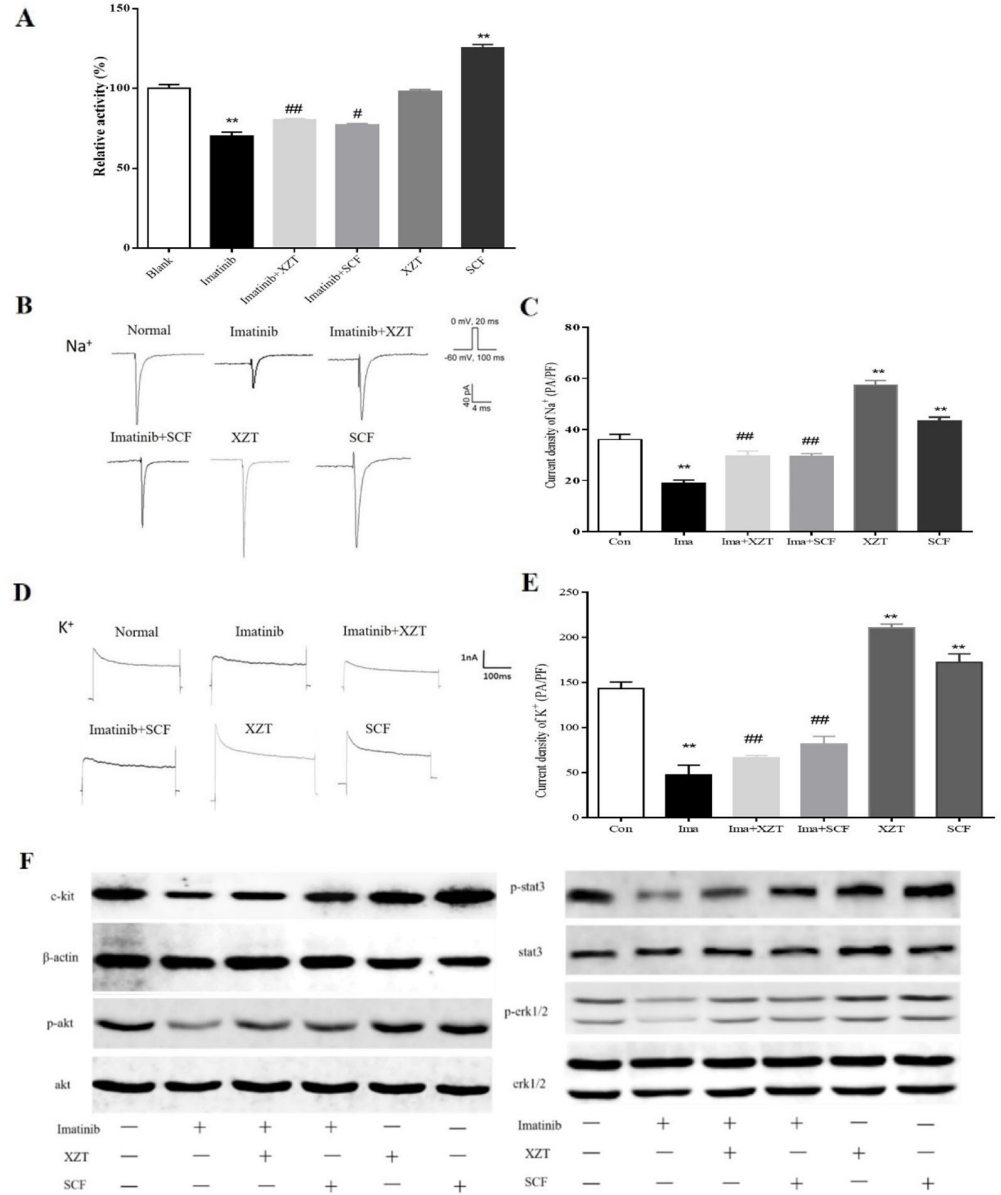
XZT regulated the SCF/c-kit pathway in ICCs *in vitro*. ICCs in the jejunum were separated from 10 g germ-free 3-day-old C57/BL6 neonatal mice. After isolation, the cells were cultured for 96 h and then incubated with (1) 100 ng/ml imatinib, (2) 100 ng/ml imatinib plus 50 ng/ml XZT, (3) 100 ng/ml imatinib plus 100 ng/ml SCF, and (4) 50 ng/ml XZT. (5) The cells were incubated with 100 ng/ml SCF for 48 h and then harvested as described. All experiments were performed at least three times using independent cell cultures. **(A)** Cell viability was detected by CCK8; **(B, D)** the whole-cell patch recording technique was used for the study of the effect of XZT on the current of Na^+^ and K^+^ channels in the ICC membrane. **(C, E)** Results of quantitative analysis of the current of the Na^+^ and K^+^ channels. **(F)** Effect of XZT on the expression of phosphorylation of c-kit, Akt, STAT3, and ERK1/2 in ICCs, as determined by western blotting. **p < 0.01 compared with the normal control group; ^#^ *p *< 0.05 and ^##^ *p *< 0.01 compared with the imatinib group.

In the concentration range of 0–100 ng/ml, SCF promoted ICCs activity and imatinib inhibited ICCs activity, both in a dose-dependent manner. XZT had no influence on ICCs activity at concentrations in the range of 0–50 ng/ml but inhibited ICCs activity at 100 ng/ml according to CCK8 (data not shown). Therefore, the final concentration in subsequent experiments was 100 ng/ml for SCF and imatinib and 50 ng/ml for XZT. The influence of XZT on the SCF/c-kit signaling pathway was comprehensively evaluated and found to exert critical effects on ICCs activity (**Figure 5A**). The expressions of c-kit, p-Akt, p-STAT3, and p-ERK1/2 in ICCs were tested using western blotting *in vitro* (**Figure 5F**). As revealed in **Figure 4C**, XZT had a reverse effect on SCF level and the phosphorylation of c-kit, Akt, STAT3, and ERK1/2 in rats with cirrhotic ascites. Moreover, XZT not only enhanced the expression of c-kit, p-Akt, p-STAT3, and p-ERK1/2 in ICCs but also antagonized the inhibition of imatinib, whereas the effect of XZT was similar to that of SCF (**Figure 5F**). The whole-cell patch clamp technique was used to study the influence of XZT on K^+^ and Na^+^ channel currents in ICCs membranes (**Figures 5B, D**), and the final results were quantitatively analyzed (**Figures 5C, E**). As disclosed in **Figures 5C, E**, XZT plays a valuable role in enhancing the current of K^+^ and Na^+^ channels in ICCs membranes as well as in reversing the inhibition effect of imatinib (**Figures 5C, E**).

## Discussion

Cirrhosis is the final stage of various chronic liver diseases and leads not only to impaired liver function but also portal hypertension. Ascites is a common complication in which cirrhosis becomes decompensated, and it results in poor life quality and high mortality. Several studies have suggested that cirrhotic ascites and gastrointestinal dysfunction might interact, but the underlying mechanism has not been revealed (Kalaitzakis et al., 2009).

CCl_4_ is a classical reagent used in model establishment of cirrhotic ascites. Relevant studies have reported that the animal model of cirrhotic ascites induced by CCl_4_ can closely duplicate the pathological features of human cirrhotic ascites, including water-sodium retention and a hyperdynamic circulation state (Lopez-Talavera et al., 1997; Domenicali et al., 2005; Ros et al., 2005). In our experiment, intraperitoneal injection with CCl_4_ was employed to induce cirrhotic ascites. To avoid high mortality caused by exposure to a heavy CCl_4_ concentration, the rats were administrated gradient concentrations of CCl_4_ in the model establishment process. A sizable fluid dark area could be seen in the abdomens of 29 rats through B ultrasound and false liver acinus caused by collagen deposition was apparent in the tissue pathology evaluated using a microscope, indicating that the cirrhotic ascites model was established successfully.

In our study, XZT obviously attenuated cirrhotic ascites in rats, as evidenced by ascites weight and measurement results through B ultrasound. Urination and fecal excretion are two principal means of exhausting water from the body. However, although conventional therapy with diuresis is commonly used, the promotion of ascites regression through diarrhea is not. In refractory ascites, bacteria translocation, disorders of proinflammatory molecules, vasoactive factors, and other disorders lead to further vasodilation of peripheral and splanchnic arteriolar vessels, resulting in hyperdynamic circulation (Hsu and Huang, 2013). In hyperdynamic circulation, the effective arterial blood volume and renal perfusion decrease and cause body resistance to dieresis. Therefore, diarrhea or the promotion of fecal water content is an alternative method for eliminating ascites. In the present study, XZT increased both 24 h urine and fecal water output, which contributed to its effect on ascites. Increases in diarrhea and fecal water content are also associated with gastrointestinal motility, and in a previous study, we observed that XZT could increase excretion and defecation, alleviating abdominal distention (Xing et al., 2012), and the results suggested that this finding was related to gastrointestinal motility. In the present study, the model rats had ascites and a delayed intestinal transit rate, but XZT ameliorated intestinal motility disorder *in vivo* and *in vitro* and increased fecal water content in the models, indicating that XZT action on ascites may be associated with the regulation of gastrointestinal motility.

Gastrointestinal motility is regulated by gastrointestinal hormones, including MTL, SP, SS, and VIP. These hormones are secreted by endocrine cells and islet cells. They stimulate smooth muscle cells and play a central role in gastrointestinal motility. In cirrhotic states, endocrinology disorder and nerve dysfunction may result in abnormal secretion and the deactivation of gastrointestinal hormones, leading to further gastrointestinal dysfunction. Thus, altering the hormone level would promote gastrointestinal motility. In the model group in our study, the MTL level was decreased and SP and SS levels were increased. XZT had a reversal effect on them, suggesting that the improvement of gastrointestinal hormone secretion could be a key action mechanism of XZT in intestinal motility.

ICCs are the pacemaker of gastrointestinal electrical activity, initiator of gastrointestinal rhythm movement, and transmitter of gastrointestinal rhythmic potential. They are widely distributed in the alimentary canal, connecting smooth muscle cells, and play a pivotal role in gastrointestinal motility (Daniel, 2004). ICC function is related to the currents of voltage-dependent ion channels and is mainly mediated by the SCF/c-Kit signaling pathway. As a type III tyrosine kinase receptor, c-kit is a specific receptor of ICCs and is mainly secreted by smooth muscle cells and liganded by SCF. Once SCF binds to a c-kit receptor in ICCs, cytoplasmic mediators are activated, including PI3K/AKT, RAS/ERK, and JAK/STAT, resulting in downstream signaling and biological effects, including with respect to gastrointestinal motility. In cases of cirrhosis, ICCs in the gastrointestinal tract reportedly decrease in number and undergo microstructural changes, which results in impaired intestinal transmission and abnormal gastrointestinal motility (Isobe et al., 1994).

To comprehensively understand the effect of XZT on the SCF/c-kit signaling pathway, which plays a critical role in ICC functions, we tested the number and viability of ICCs and their electronic activities, in particular the signaling of mediator expression and phosphorylation, *in vivo* and *in vitro* with the inhibitor imatinib as the control. Our results indicated decreased c-kit expression and cell viability and downregulated current of sodium and potassium ions in ICCs in the model rats. XZT appeared to increase c-kit expression, enhance the ICC cell number and viability, and promote voltage-dependent sodium and potassium current. In addition, XZT countered the influence of imatinib, an inhibitor of tyrosine kinase, on ICC activities, such as the movement of sodium and potassium ions. Finally, XZT improved the expression and phosphorylation of mediators such as ERK1/2 and STAT3 in SCF/c-kit signaling. The results indicate that XZT at least partially restores ICCs functions, mainly through the regulation of the SCF/c-kit signaling pathway, which is closely associated with its regulation of gastrointestinal motility in rats with cirrhotic ascites. Our findings not only reveal the main molecular mechanism of XZT on gastrointestinal motility in cirrhosis but also provide a novel method for the treatment of cirrhotic ascites.

## Conclusion

XZT alleviated ascites and improves gastrointestinal motility disorders in cirrhotic rats. XZT action on gastrointestinal motility may be associated with its effect on ascites, and the mechanism of XZT on gastrointestinal motility was mainly regarded as regulating SCF/c-kit pathway in ICCs and gastrointestinal hormone secretion.

## Data Availability Statement

The raw data supporting the conclusions of this manuscript will be made available by the authors, without undue reservation, to any qualified researcher.

## Author Contributions

QZ and FX performed the experiments. QZ, FX, and YT analyzed the data and wrote the manuscript. KH and HL assisted with the animal experiment. YP assisted with the cell culture. CL and NF designed the study, NF made the XZT extract, and CL made critical revisions to the manuscript. CL and NF serve as the corresponding authors for the project.

## Funding

This work was supported by grants from the National Natural Science Foundation of China (No. 81473479, No. 81573619, and No. 81730109) and the “Three-Year Action Plan” for Development of TCM in Shanghai (Grant No. 16CR1026B).

## Conflict of Interest

The authors declare that the research was conducted in the absence of any commercial or financial relationships that could be construed as a potential conflict of interest.
