# Supplementary material for: Xiaozhang Tie Improves Intestinal Motility in Rats With Cirrhotic Ascites by Regulating the Stem Cell Factor/c-kit Pathway in Interstitial Cells of Cajal
Source: Front Pharmacol. 2020 Feb 4;11:1. doi: 10.3389/fphar.2020.00001 (PMC7011082; doi:10.3389/fphar.2020.00001)
Supplement: Supplementary file 1 [file DataSheet_1.doc]

**Supplementary methods for XiaozhangTie Quality of Control**

*XZT extraction methods*

XZT was extracted using an ultrasonic-assisted extraction method as previously described.^1^ *XZT* was cut into small pieces and mixed with 60 mL of 80% (v/v)methanol aqueous solution into a volumetric flask. The bottles were then closed and placed in the ultrasonic bath with the temperature maintained at 20°Cfor 30 min (40 kHz, SB-5200D, Ningbo Scientz Technology Co. Ltd, Ningbo, China).The prepared extracts were then transferred to 10 mL centrifuge tubes and centrifuged at 6,000 g for 10 min. Then, the supernatants were filtered by a 0.22μm syringe filter and stored at 4°C until further analyzed.

XZT essential oil was obtained by a hydro-distillation method as described in the pharmacopoeia of the People’s Republic of China.^2^ *XZT* was cut into small pieces, soaked in water (300 mL) for 12 h, and then distilled at the temperature of 200°C for 5 h using a Clevenger-type apparatus. The yellowish colored essential oils were collected and stored in sealed vials at 4°C until the time of analysis.

*UPLC-QTOF-MS/MS experimental parameters in the compositional analysis*

The compositional analysis of *XZT* extracts was performed on a Waters Acquity Ultra-Performance LC-Synapt G2 Q/TOF system (Waters Corporation, Milford, MA, USA). The column was a 50 mm × 2.1 mm, 1.7 μm, Syncronis C18 reverse-phase column (ThermoFinnigan, San Jose, CA, USA) and the temperature was maintained at 30°C. The mobile phase consisted of acetonitrile (A) and 5mM ammonium acetate (B) with a gradient as follows: 0-3 min, 5%-15% A; 3-5 min, 15%-23% A; 5-7 min, 23%-40% A; 7-17 min, 40%-65% A; 17-19 min, 65%-70% A; 19-24 min, 70%-95% A; 24-32 min, 95% A. The flow rate was raised from0.2 mL/min (0-25 min) to 0.5 mL/min (25-32 min).The injection volume was 5 μL.

The QTOF Premier mass spectrometer was operated on a fullscanoperation in both positive and negative ionization modes using an electrospray ionization (ESI )source. Capillary voltage was maintained at +3 kV and -2.5kV in the positive and negative modes, respectively. Conevoltage was set to+30Vfor the positive mode and -25 V for the negative mode. The source temperature was 120°C and the desolvation gas temperature was 350°C. Nitrogenwas used as both cone gas (50 L/h) and desolvation gas (800 L/h), and argon was used as collision gas. For accurate mass analysis, the mass spectrometer was monitored by intermittent injection of the lock mass leucine-enkephaline([M+H]^+^ = 556.2771 m/z; [M-H]^-^ = 554.2615 m/z) at a concentration of 2 ng/mL in real time. Data were collected in centroid mode from 100 to 1,000m/z. Meanwhile, the MS/MS fragment information was obtained using a collision energy ramp from 10 eV to 30 eV.

Putative identifications for the resulting ion list were obtained through mass-based search against three databases: PubChem (http://pubchem.ncbi.nlm.nih.gov), ChemSpider(http://www.chemspider.com), and mzCloud (http://www.mzcloud.org).The mass tolerance in the database search was set to 5 ppm and 2.2 mDa. Furthermore, the identified compounds were verified by comparing their MS spectra and retention time with the commercially available standards.


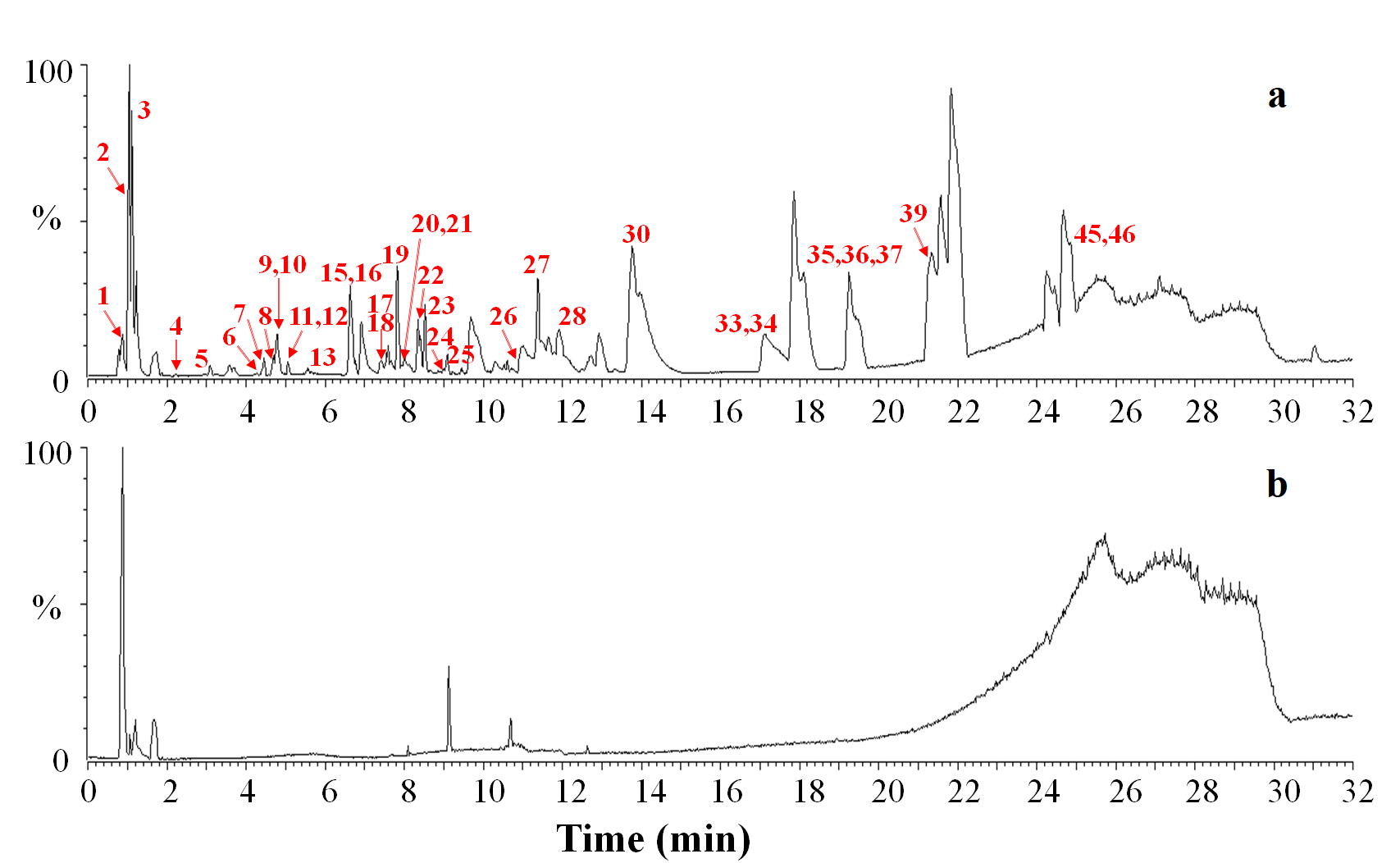


**Fig. 1** Base peak chromatograms of Xiaozhang Tie extracts obtained by UPLC-QTOF-MSin the positive ion mode.(a, Xiaozhang Tie; b, blank cataplasm)

| **The Formula of Xiaozhang Tie and Quality of Control (one dose)** | | | | |
| --- | --- | --- | --- | --- |
| **Drug name** | plant sources | Medicinal parts | Dosage/g | Quality control |
| **Dahuang** | Rheum palmatum L. | rhizoma | 1 | **Emodin >=0.42 mg** |
| **Laifuzi** | Raphanus sativus L. | seed | 1 |  |
| **Gansui** | Euphorbia kansui T. N. Liou ex T. P. Wang | root | 1 |  |
| **Chenxiang** | Aquilaria sinensis (Lour.)Gilg | stem | 0.2 |  |
| **Dingxiang** | Eugenia caryophyllata Thunb. | flower bud | 1 |  |
| **borneolum syntheticum** |  |  | **0.04** | **Borneol >=18.0 mg** |
| **artificial moschus** |  |  | 0.004 |  |

Reference

1. Xing, F., Y. Tan, G. J. Yan, J. J. Zhang, Z. H. Shi, S. Z. Tan, N. P. Feng & C. H. Liu. Effects of Chinese herbal cataplasm Xiaozhang Tie on cirrhotic ascites. J Ethnopharmacol, 2012, 139, 343-9.
2. Zhang, K., Zhang, Y., Li, N., Xing, F., Zhao, J., Yang, T., Liu, C., and Feng, N. (2019). An herbal-compound-based combination therapy that relieves cirrhotic ascites by affecting the L-arginine/nitric oxide pathway: A metabolomics-based systematic study. J Ethnopharmacol, 2019, 241, 112034.
